# Tensile force-induced PDGF-BB/PDGFRβ signals in periodontal ligament fibroblasts activate JAK2/STAT3 for orthodontic tooth movement

**DOI:** 10.1038/s41598-020-68068-1

**Published:** 2020-07-09

**Authors:** Yuqin Jin, Liang Ding, Zhuang Ding, Yong Fu, Yuxian Song, Yue Jing, Qiang Li, Jianyun Zhang, Yanhong Ni, Qingang Hu

**Affiliations:** 1 Department of Orthodontics, Nanjing Stomatological Hospital, Medical School of Nanjing University, Nanjing, China; 2 Central Laboratory of Stomatology, Nanjing Stomatological Hospital, Medical School of Nanjing University, No. 30 Zhongyang Road, Nanjing, 210008 China; 3 Department of Oral and Maxillofacial Surgery, Maxillofacial Surgery, Nanjing Stomatological Hospital, Medical School of Nanjing University, No. 30 Zhongyang Road, Nanjing, 210008 China

**Keywords:** Cell biology, Molecular biology

## Abstract

Orthodontic force-induced osteogenic differentiation and bone formation at tension side play a pivotal role in orthodontic tooth movement (OTM). Platelet-derived growth factor-BB (PDGF-BB) is a clinically proven growth factor during bone regeneration process with unclear mechanisms. Fibroblasts in periodontal ligament (PDL) are considered to be mechanosensitive under orthodontic force. Thus, we established OTM model to investigate the correlation between PDGF-BB and fibroblasts during bone regeneration at tension side. We confirmed that tensile force stimulated PDL cells to induce osteogenic differentiation via Runx-2, OCN up-regulation, and to accelerate new bone deposition along the periodontium and the alveolar bone interface. Interestingly, PDGF-BB level was remarkably enhanced at tension side during OTM in parallel with up-regulated PDGFRβ+/α-SMA+ fibroblasts in PDL by immunohistochemistry. Moreover, orthodontic force-treated primary fibroblasts from PDL were isolated and, cultured in vitro, which showed similar morphology and phenotype with control fibroblasts without OTM treatment. PDGFRβ expression was confirmed to be increased in orthodontic force-treated fibroblasts by immunofluorescence and flow cytometry. Bioinformatics analysis identified that PDGF-BB/PDGFRβ signals were relevant to the activation of JAK/STAT3 signals. The protein expression of JAK2 and STAT3 was elevated in PDL of tension side. Importantly, in vivo, the treatment of the inhibitors (imatinib and AG490) for PDGFRβ and JAK–STAT signals were capable of attenuating the tooth movement. The osteogenic differentiation and bone regeneration in tension side were down-regulated upon the treatment of inhibitors during OTM. Meanwhile, the expressions of PDGFRβ, JAK2 and STAT3 were inhibited by imatinib and AG490. Thus, we concluded that tensile force-induced PDGF-BB activated JAK2/STAT3 signals in PDGFRβ^+^ fibroblasts in bone formation during OTM.

## Introduction

Mechanical forces are integral to bone homeostasis and bone regeneration by driving cell differentiation^1^. It was reported that the mechanical force applied during mandible distraction osteogenesis promoted endogenous bone formation across a mechanically controlled environment^2,3^. During the process of OTM, the appropriate application of mechanical force promotes alveolar bone remodeling, which consists of bone resorption at compression side and bone formation at tension side of the alveolar bone^4,5^.

Periodontal ligament (PDL) is a multifunctional fibrous tissue that physically connects the tooth root to its surrounding alveolar bone^6,7^. Mechanosensitive cells of the PDL, the PDL fibroblasts, are able to transduce mechanical strain into intracellular signals, which leads to the remodeling of both the periodontium and alveolar bone^8,9^. Under cyclic strain, PDL fibroblasts in vitro increased their osteogenic gene expression^10^. When the mechanical force is applied in vivo, periodontal ligament cells on the tension side synthesize and secrete cytokines, growth and differentiation factors, such as runt-related transcription factor-2 (Runx-2), osteoprotegerin (OPG), bone morphogenetic proteins (BMPs), to regulate bone synthesis^4,11,12^.

Platelet-derived growth factor-BB (PDGF-BB) was originally identified in platelets, a member of the PDGF family, has been recognized as a key regulator in wound healing and tissue repair^13,14^. We previously demonstrated that recombinant human PDGF-BB (rhPDGF-BB) promoted adipose derived stem cells (ADSCs) proliferation and osteogenic differentiation and suppressed adipogenic differentiation in vitro via ERK pathway and that ADSCs associated with rhPDGF-BB could be a promising tissue-engineered construct for craniofacial bone regeneration in vivo^15^. PDGF-BB binds to the extracellular domains of PDGF receptors including PDGFRα and PDGFRβ, leading to the activation of multiple signaling pathways^16^. However, it is not clear whether PDGF-BB/PDGFR signals plays a role in bone regeneration at tension side during OTM. It is well known that PDGFRβ is almost exclusively expressed in fibroblasts^17,18^, but whether fibroblasts in PDL response to PDGF-BB, and then participate in bone regeneration at tension side during OTM remains elusive.

Therefore, we established a rat orthodontic tooth movement model to investigate the expression of PDGF-BB and the response of PDGFRβ^+^ fibroblasts at tension area. Moreover, we cultured the primary orthodontic force-induced PDL fibroblasts to evaluate the expression of PDGFRβ. We also explored the PDGFRβ-related signals through bioinformatics analysis, and finally verified the probable pathway in vivo via OTM model. Moreover, the local administration of the inhibitors of PDGFRβ and JAK/STAT signals during OTM indeed restricted tooth movement compared with the vehicle-injected OTM group by down-regulating the osteogenic differentiation and new bone formation via suppressing the PDGFRβ/JAK2/STAT3 signals. These results demonstrated that tensile force-induced PDGF-BB activated JAK2/STAT3 signals in PDGFRβ^+^ fibroblasts in bone formation during OTM.

## Results

### Orthodontic tooth movement and micro-CT analysis

**Figure 1:**
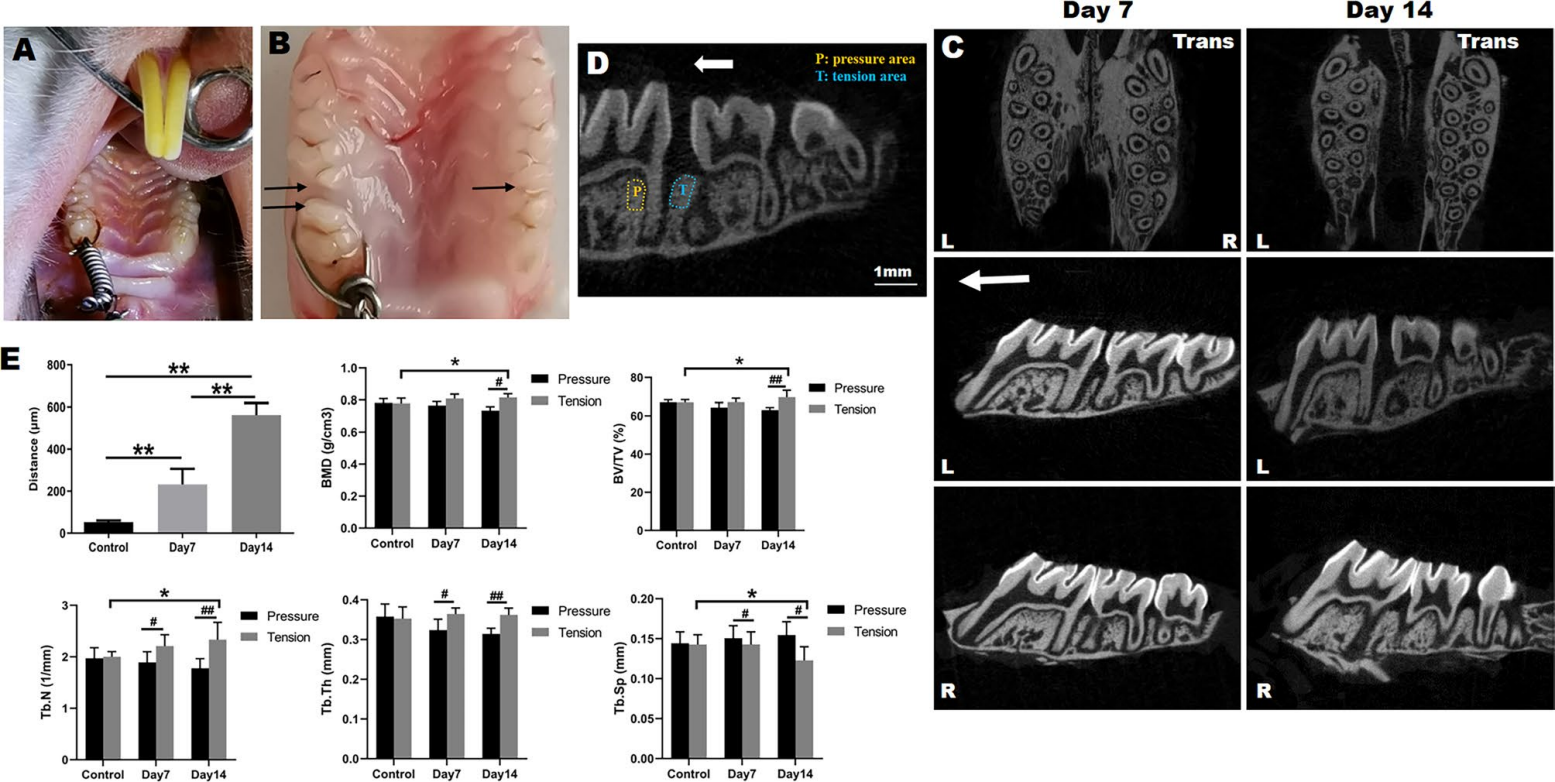
Orthodontic tooth movement and representative three-dimensional images of maxillae in SD rats. (**A**) Orthodontic appliance placement. A coiled-spring was placed between the left maxillary first molar and the maxillary incisors with cured resin on the incisors. (**B**) A representative image of tooth movement mesially at day 7. Note the amount of space visible between the first and second molar. The distance was indicated by black arrows. (**C**) Representative three-dimensional images of the maxilla at day 7 and day 14 of OTM. The white arrow indicated the direction of force. *Trans* transverse, *L* left, *R* right. (**D**) The region of interest (ROI) was defined as the zone of alveolar bone at the distal coronal one-third area of the distal root (the tension area, T) and the mesial coronal one-third area (the pressure area, P) for Micro-CT analysis. The white arrow indicated the direction of force. (**E**) Analysis of the distance of tooth mesial movement and microstructural parameters of alveolar bone in control group and at day 7 and 14 of OTM. *BMD* bone mineral density, *BV/TV* bone volume/total volume, *Tb.Sp* trabecular separation, *Tb.Th* trabecular thickness, *Tb.N* trabecular number. Each column represents the mean value of triplicate experiments. *p < 0.05, **p < 0.01; ^#^*p* < 0.05, ^##^*p* < 0.01 comparison between tension area and pressure area in each group, n = 8/group.

We performed OTM of the maxillary left first molar with a widely-used model in SD rats (Fig. 1A)^19^. The distance between the first and second molars was increased in a time dependent manner, with a relatively rapid increasement at day 14 (Fig. 1B,C). And there were statistical significances among the three groups (Fig. 1E). The morphology of the maxillary was reconstructed using Micro-CT from transversal and sagittal views in control group and at day 7 and 14 of OTM (Fig. 1C). The alveolar bone at the distal coronal one-third area of the distal root was the tension area (T) and the pressure area (P) was the mesial coronal one-third area. As shown in Fig. 1D, the tension area and pressure area were selected as the region of interest (ROI) for Micro-CT analysis. To evaluate the alveolar bone mass and microarchitectural changes in tension area and pressure area of the left maxillae during OTM and the right untreated maxillae (Control group), we analyzed microstructure parameters, named BMD, BV/TV, Tb.N, Tb.Th and Tb.Sp (Fig. 1E). In control group, all the five bone parameters between the pressure area and tension area showed the similar level, without statistical difference. However, after orthodontic force treatment, BMD, BV/TV, Tb.N, Tb.Th in tension area were obviously higher than that in pressure area, especially at day 14 of OTM, conversely, Tb.Sp in tension area revealed a marked decrease. When compared with the tension area among the three groups, it was seen that BMD, BV/TV, Tb.N and Tb.Th increased in tension area from day 7 to day 14 compared with that of control group during OTM, while Tb.Sp decreased significantly at day 14 of OTM.

### The morphological changes of periodontal ligament and new bone formation induced by tension force during OTM

**Figure 2:**
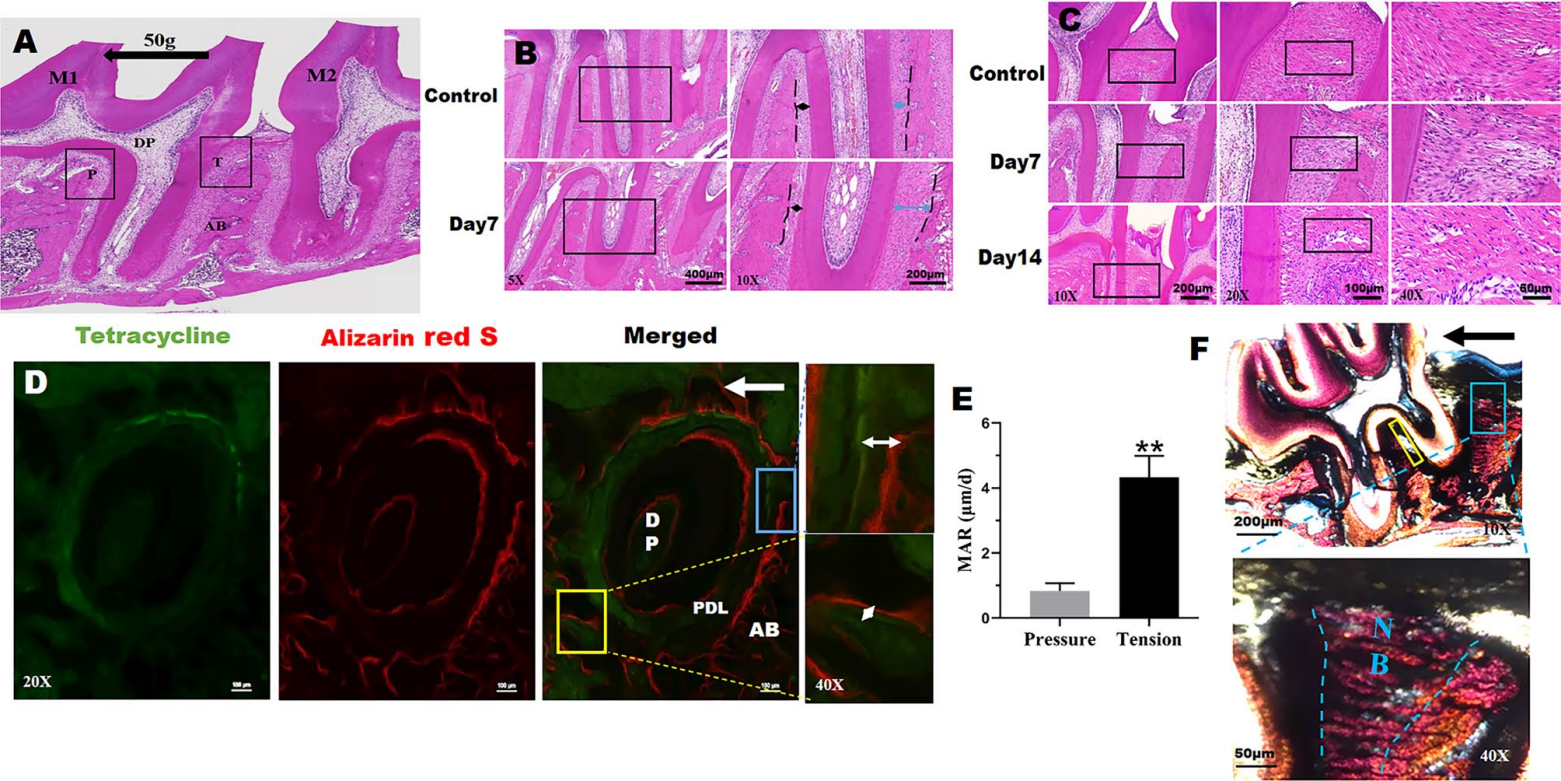
Tension force induced the periodontal ligament change and new bone formation in the tension area during OTM. (**A**) Schematic graph illustrating the region of interest for histological analysis during OTM. The distal coronal one-third area of the distal root was the tension area (T), and the pressure area (P) was the mesial coronal one-third area. *M1* the left maxillary first molar, *M2* the left maxillary second molar, *AB* alveolar bone, *DP* dental pulp; The black arrow indicated the direction of force. (**B**) Representative H-E staining images showed the width of periodontal ligament changes in the tension (blue arrows) and compression side (black arrows) in control group and at day 7 of OTM. (**C**) H-E staining showed the morphology change of periodontal ligament at tension side in Control group, and at day 7 and 14 of OTM. The boxed regions are shown at a higher magnification in their corresponding right figures. (**D**) Sequential fluorescence labeling observations at day 28 of OTM. Green and red represent labeling by Tetracycline (day 7) and Alizarin Red S (day 14), respectively. The white two-way arrows indicated the newly formed bone. (**E**) The statistics of mineral apposition rate (MAR) at tension side and pressure side at day 28 of OTM. (**F**) Van Gieson staining at day 28 of OTM. The black arrow indicated the direction of force. The blue boxed region (tension area) and yellow boxed region (pressure area) were shown at a higher magnification in their corresponding figures, respectively. *AB* alveolar bone, *DP* dental pulp, *PDL* periodontal ligament, *NB* new bone. Each column represents the mean value of triplicate experiments. **p < 0.01, comparison between Tension group (tension side) and Pressure group (pressure side), n = 8/group.

In this study, the distal coronal one-third area of the distal root of the first molar was selected as the tension area (T) for histological analysis, and conversely, the pressure area (P) was the mesial coronal one-third area (the direct opposite area) of that (Fig. 2A). Representative H-E staining images showed the changes of the width of periodontal ligament in the tension side (blue arrows) and compression side (black arrows) in control group and at day 7 of OTM (Fig. 2B). In control group, the width of PDL on both sides of the distal root was similar. PDL in the pressure zone at day 7 of OTM was extremely contracted under the compressive stress. While, PDL in the tension side was stretched, the width of which was much larger than the pressure side (Fig. 2B). Periodontal ligament fibroblasts on the tension side arranged in disorder at day 7, by contrast, fibroblasts arranged in order in control group (Fig. 2C). Moreover, in the tension zone, the PDL fibroblasts appeared to be elongated at day 14 of OTM (Fig. 2C), which was in line with previous studies^20^. Polychrome sequential labeling with fluorochromes is a standard technique for labeling the mineralized tissue and assess the time course of new bone formation and mineralization in vivo^21,22^. Tetracycline (TE) and Alizarin Red S (AL) were injected at day 7 and day 14 of OTM, respectively. Then the newly formed bone was analyzed by confocal microscopy, as shown in Fig. 2D. The total area of AL on the distal coronal one-third area of the distal root (tension area) was much higher than that on the mesial coronal one-third area of that (pressure area), and the same tendency was seen with TE. Moreover, it can be seen from the right magnified figures that the width of the newly formed bone (white two-way arrow) on the tension side was much bigger than that on the compressive side (Fig. 2D). And the distance between the fluorescence bands reflects the deposition rate of bone mineralization^23^. As shown in Fig. 2E, bone mineral apposition rate (MAR) was significantly higher in tension area than that in pressure area. To further confirm the aforementioned sequential fluorescence labeling results in tension area, Van Gieson staining was used to detect new bone formation in undecalcified sections at day 28 of OTM (Fig. 2F). A large amount of new bone was deposited along the periodontium and the alveolar bone interface in the tension area compared with the pressure area at day 28. These results confirmed that new bone was deposited in the tension area by tensile stress during OTM.

**Figure 3:**
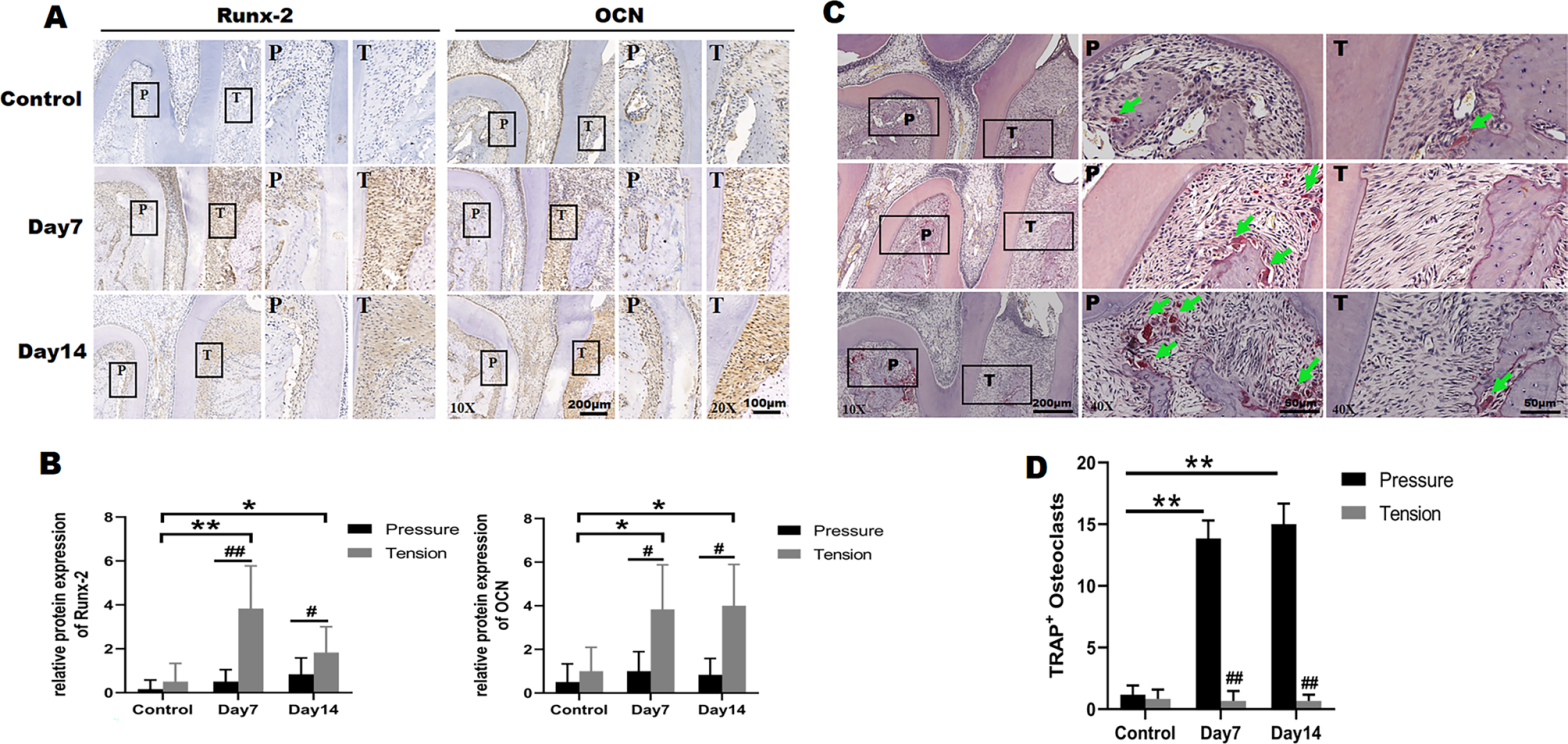
Osteogenesis at tension side and osteoclastogenesis at compression side during OTM. (**A,B**) Immunohistochemistry analysis for Runx-2 and OCN in the pressure area and tension area during OTM (**A**) and the semi-quantitative analysis (**B**). The boxed regions are shown at a higher magnification in their corresponding right figures. *P* pressure area, *T* tension area. (**C,D**) TRAP staining for osteoclasts (**C**) and the number of TRAP-positive osteoclasts (**D**) at pressure area and tension area in three groups. The boxed regions are shown at a higher magnification in their corresponding right figures. The green arrows indicated osteoclasts. *P* pressure area, *T* tension area. Each column represents the mean value of triplicate experiments. ^#^*p* < 0.05, ^##^*p* < 0.01 comparison between tension area and pressure area in each group; *p < 0.05, **p < 0.01, n = 8/group.

To understand the molecular mechanism underlying the changes in bone formation during OTM, we analyzed the expression of Runx-2 and OCN via immunohistochemistry (IHC) in the control group and at day 7 and 14 of OTM (Fig. 3A). Runx-2 and OCN, which reflected osteogenic differentiation, were significantly up-regulated in tension area by tension force than that in pressure area both at day 7 and day 14 of OTM, respectively. In contrast, Runx-2 and OCN were weakly expressed in both areas of control group. Among the three groups, the expression of Runx-2 and OCN in tension area increased significantly in experimental groups compared with the control group, and the staining intensity of Runx-2 in tension side was more pronounced at day 7 than day 14 (Fig. 3B). Besides, the expression of OCN at tension side peaked at day 14 of OTM. The up-regulation of Runx-2 and OCN at tension side indicated that tensile force induced osteogenic differentiation and later bone formation during OTM. Meanwhile osteoclasts activity in the pressure area is also critical to the tooth movement rate, which was evaluated by TRAP staining. As shown in Fig. 3C, there were almost no osteoclasts in both areas of the control group. However, the number of TRAP-positive osteoclasts was significantly increased in the pressure zone at day 7 and day 14 of OTM compared with the pressure area of control group (Fig. 3C,D). Furthermore, there were obviously more osteoclasts in the pressure side versus the tension side in two experimental groups. These results suggested that osteoclastogenesis and bone resorption in pressure side were stronger than that in tension side.

### PDGF-BB responsive-fibroblasts are up-regulated by orthodontic force

**Figure 4:**
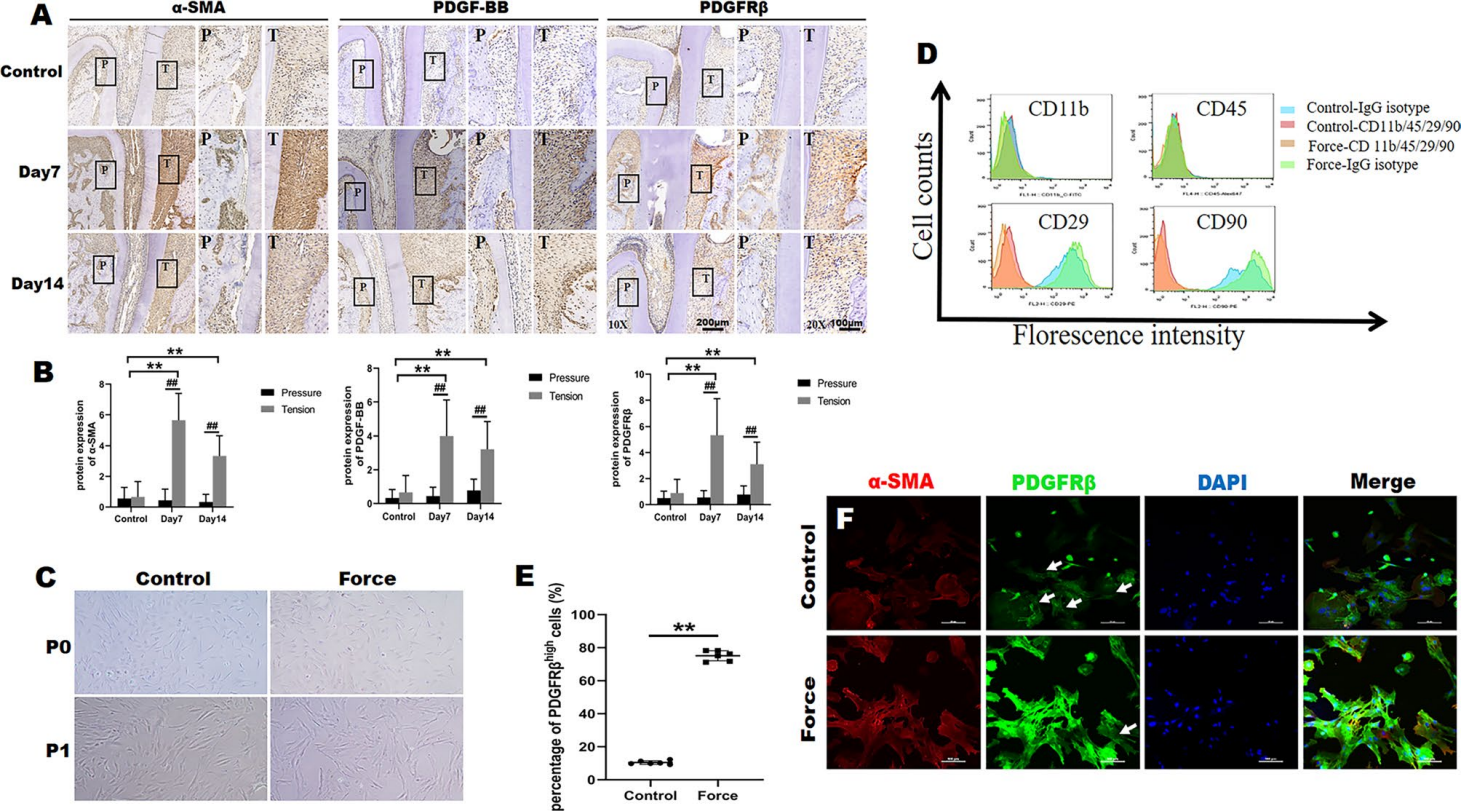
PDGF-BB responsive-fibroblasts are up-regulated by orthodontic force. (**A**) Protein expression of α-SMA, PDGF-BB, PDGFRβ was measured by immunohistochemistry (IHC) in Control group and at day 7 and day 14 of OTM. The boxed regions are shown at a higher magnification in their corresponding right figures. *P* pressure area, *T* tension area. (**B**) The semi-quantitative analysis for IHC. (**C**) Cell morphology of normal PDL fibroblasts and orthodontic force-induced PDL fibroblasts. (**D**) Identification of PDL fibroblasts by flow cytometry. Identification of cell surface markers CD11b, CD45, CD29 and CD90. (**E**) Percentage of PDGRRβ^high^ PDL fibroblasts in Control group and Force group. **p < 0.01, n = 6/group. (**F**) Representative immunofluorescence images stained of α-SMA (red), PDGFRβ (green), and DAPI (blue, nuclei) in normal PDL fibroblasts and orthodontic force-induced PDL fibroblasts. The arrows indicated low-expressed PDGFRβ PDL fibroblasts. Each column represents the mean value of triplicate experiments. ^##^*p* < 0.01 comparison between tension area and pressure area in each group, **p < 0.01 comparison with the tension area among three groups, n = 8/group.

α-SMA is a characteristic marker of fibroblasts^24–26^. Immunohistochemistry analysis found that α-SMA increased notably in tension area at day 7 compared with the tension area of control group, and then decreased slightly at day 14 (Fig. 4A,B). While the number of α-SMA positive fibroblasts in the pressure area were far less than that in the tension area during OTM. In addition, PDGF-BB and PDGFRβ expression showed remarkable up-regulation as early as day 7 at tension side during orthodontic tooth movement compared with the tension area of control group (Fig. 4A,B). And the expression of PDGF-BB and PDGFRβ in tension area was significantly higher than the pressure area at day 7 and day 14 of OTM, respectively. Subsequently, orthodontic force-treated primary fibroblasts (Force fibroblasts) and normal fibroblasts from PDL were isolated and cultured in vitro for investigation. The Force fibroblasts and normal fibroblasts showed a similar long fusiform cell morphology (Fig. 4C). And molecular surface markers of PDL fibroblasts were examined by flow cytometry (Fig. 4D). These two kinds of fibroblasts present a high expression of CD29 and CD90, whereas the expression of CD11b and CD45 was rarely observed (Fig. 4D). Moreover, we found that approximately 75% of total PDL cells in Force group were high-expressed PDGFRβ, while there was only about 10% in Control group (Fig. 4E). This result was consistent with that of IHC for PDGFRβ. Furthermore, PDGFRβ expression was confirmed to be increased in orthodontic force-treated fibroblasts by IF (Fig. 4F). It was shown that most of the fibroblasts in Force group were high-expressed PDGFRβ, while most of that in control group were low-expressed PDGFRβ. The expression of α-SMA was also up-regulated in Force group (Fig. 4F). In conclusion, the PDL fibroblasts in Force group with high-expressed of PDGFRβ mostly come from the tension area. We can define the origin of PDL fibroblasts in Force group was mainly from the tensile area.

### PDGF-BB/PDGFRβ might regulate bone formation under tensile force through the JAK2/STAT3 pathway

**Figure 5:**
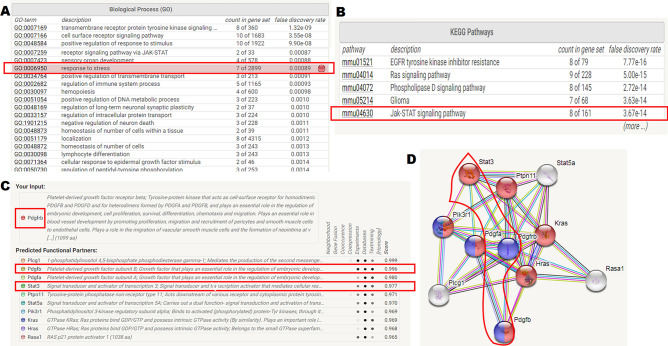
Bioinformatics analysis of conceivable factors interacted with PDGFRβ. (**A**) Biological process (GO). (**B**) KEGG pathways. (**C,D**) PDGFRβ predicted functional partners analyzed by STRING. *GO* Gene Ontology, *KEGG* Kyoto Encyclopedia of Genes and Genomes.

**Figure 6:**
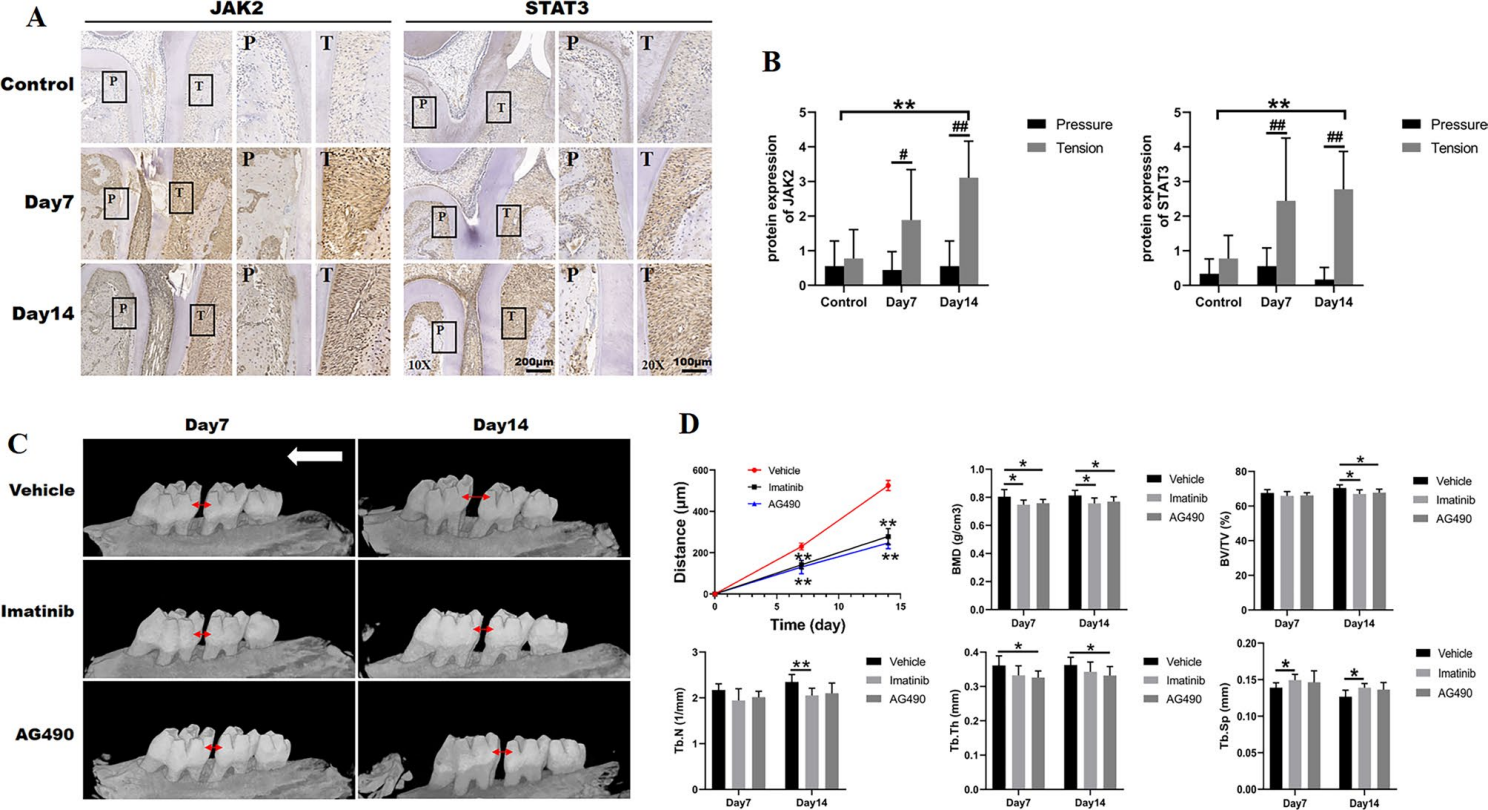
PDGFRβ/JAK2/STAT3 pathway might regulate bone formation in tension side during OTM. (**A**) Protein expression of JAK2, STAT3 was measured by immunohistochemistry (IHC) in Control group and at day 7 group and day 14 group. The boxed regions are shown at a higher magnification in their corresponding right figures. *P* pressure area, *T* tension area. (**B**) The semi-quantitative analysis for IHC. (**C**) Three-dimensional sagittal views of the left maxillae in Vehicle, Imatinib and AG490 groups at day 7 and day 14 of OTM. Note the interdental distance visible between the first and second molar. The red two-way arrows indicated the distance between the first and second molars during OTM. The white arrow indicated the direction of force. (**D**) Analysis of the distance of tooth mesial movement and microstructural parameters of alveolar bone in tension area of Vehicle, Imatinib and AG490 groups at day 7 and 14 of OTM. *BMD* bone mineral density, *BV/TV* bone volume/total volume, *Tb.N* trabecular number, *Tb.Th* trabecular thickness, *Tb.Sp* trabecular separation. Each column represents the mean value of triplicate experiments. *p < 0.05, **p < 0.01 compared with the Vehicle group at day 7 or day 14 of OTM, n = 8/group.

In order to explore the underlying mechanism of periodontal ligament fibroblasts in bone formation, mediated by PDGF-BB/PDGFRβ, we utilized bioinformatics analysis to investigate the biologic characteristics of PDGFRβ shared by STRING. The biological process (GO) focused on response to stress function (Fig. 5A), and the KEGG pathway predicted the potential JAK–STAT signaling pathway with a less false discovery rate (Fig. 5B). Based on the above mentioned, we speculated that STAT3 may be involved in the PDGFRβ regulation (Fig. 5C,D). Bioinformatics analysis identified that PDGF-BB/PDGFRβ signals were relevant to the activation of JAK/STAT3 signaling pathways, which was also consistent with the findings in OTM model. As shown in Fig. 6A,B, JAK2 and STAT3 displayed increase at day 7 and became significant at day 14 of OTM in tension side compared with the same area of control group. On contrary, the expression of JAK2 and STAT3 in pressure area of the experimental groups was obviously weaker than that in tension area during OTM. In order to further verify whether the PDGFRβ/JAK2/STAT3 signaling pathway was involved in OTM, we locally injected the related inhibitors in vivo. It was found that local delivery of imatinib or AG490 attenuated the mesial movement of the first molar compared with the Vehicle group at day 7 (Fig. 6C,D). By day 14, there was a substantial decrease of mesial molar movement in both Imatinib group and AG490 group (Fig. 6C,D). To evaluate the alveolar bone mass and microarchitectural changes in the tension area upon the local administration of inhibitors during OTM, we analyzed Micro-CT parameters. And it revealed that BMD, BV/TV, Tb.N, Tb.Th in tension area were decreased in Imatinib group and AG490 group in comparison with the same area of the Vehicle group (Fig. 6D). BMD and Tb.Th showed marked down-regulation as early as day 7 in inhibitor-treated groups, whereas BV/TV, Tb.N displayed a slight decrease at day 7, which became statistically significant at day 14 (Fig. 6D). Meanwhile, Tb.Sp was increased upon the treatment of inhibitors compared with the Vehicle group, and there were statistical differences between Vehicle group and Imatinib group at day 7 and day 14 of OTM (Fig. 6D). These results indicated that the treatment of inhibitors during OTM inhibited the new bone formation at tension side.

**Figure 7:**
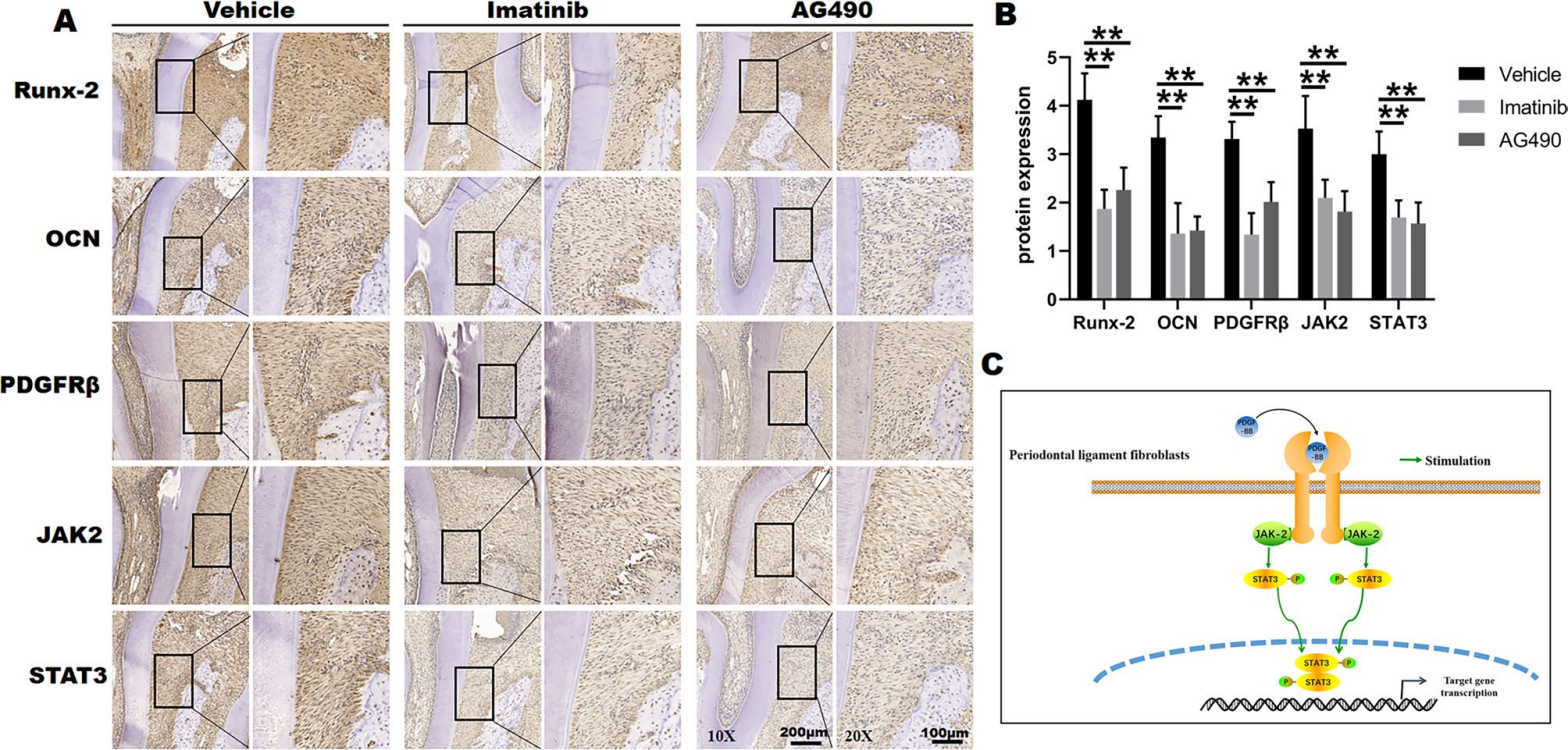
The expressions of PDGFRβ-related pathway during OTM. (**A**) Protein expression of Runx-2, OCN, JAK2 and STAT3 was measured by immunohistochemistry (IHC) in tension area of Vehicle, Imatinib and AG490 groups at day 7 of OTM. (**B**) The semi-quantitative analysis for IHC. (**C**) Schematic graph illustrates the mechanism of the PDGF-BB/PDGFRβ/JAK-2/STAT3 signal axis regulating bone formation under tensile force during OTM. Each column represents the mean value of triplicate experiments. **p < 0.01 compared with the Vehicle group at day 7 of OTM, n = 8/group.

By IHC, it was found that the expression of Runx-2 and OCN in tension area was significantly down-regulated in Imatinib group and AG490 group compared with the Vehicle group at day 7 of OTM (Fig. 7A,B). The results indicated that upon the administration of inhibitors of PDGFRβ and JAK/STAT, the osteogenic differentiation in tension side was reduced, which was consistent with the Micro-CT quantitative analysis. In addition, the expression of PDGFRβ, JAK2 and STAT3 was notably decreased after Imatinib or AG490 treatment, showing the same trend as the expression of Runx-2 and OCN (Fig. 7A). And there were statistical differences of PDGFRβ, JAK2 and STAT3 between Vehicle group and Imatinib group or AG490 group, respectively (Fig. 7B). Consequently, these results indicated that tensile force-induced PDGF-BB activated JAK2/STAT3 signals in PDGFRβ^+^ fibroblasts in regulating bone formation during OTM (Fig. 7C).

## Discussion

Bone formation, consisting of osteoid formation, maturation and secondary mineralization, is a slow process, whereas bone absorption is a relatively fast process. In our study, it was shown that the bone mineral density of the alveolar bone in tension side was slightly increased at day 7 of OTM compared to the same area of the control group, but became more pronounced at day 14. The OTM undergo 3 stages, and initial stage is characterized by tooth displacement in the periodontal ligament space within its bone socket. Stage two is characterized by the formation of necrosis and hyalinization in response to compression stress at pressure side. While in stage 3, there is tooth movement mediated by bone remodeling through the agency of osteoclasts and osteoblasts^4,27,28^. And at day 28 of OTM, it was shown that there was massive newly formed bone at tension side compared with the pressure area from the histological observation, which was consistent with the Micro-CT analysis. However, the mechanism of orthodontic force-induced bone formation at tension side during OTM is unclear.

Orthodontic force is transmitted through the PDL to the supporting alveolar bone and lead to bone remodeling. The PDL contains a variety of cell populations, consisting of fibroblasts, osteoblasts, osteoclasts, cementoblasts, endothelia cells and stem cells^29,30^, thus enabling the PDL to perform supportive, remodeling, and homeostatic functions. Among these cells, fibroblasts are high dominant cellular components in PDL^6^, which surround the tooth roots and connect to the alveolar bone. Fibroblasts in PDL are considered to be the first recipients of mechanical strain and mechanoresponsive^6^. We here found that the number of α-SMA^+^ fibroblasts was significantly increased at tension side and peaked at day 7, indicating the periodontal ligament fibroblasts changed from relatively quiescent state to proliferating state under the tensile stress, which was consistent with previous studies^31^. However, the transduction mechanisms of mechanical force from fibroblasts to the initiation of bone remodeling are far from known. Recent studies demonstrated that fibroblasts exposed to tensile strain in vitro induced the expression of ephrin-B2 via a FAK-, Ras-, ERK1/2-, and SP1-dependent pathway^6^. Previous in vitro studies showed that augmented mechanical stress-mediated intracellular Ca^2+^ oscillations in human PDL fibroblasts enhanced the production and release of bone regulatory signals via Rankl/OPG and the canonical Wnt/β-catenin pathway as an early process in tooth movement-initiated alveolar bone remodeling^8^. We here found that PDGF-BB and PDGF-BB responsive-fibroblasts showed remarkable up-regulation as early as day 7 at tension side in vivo, indicating the potential role of periodontal ligament fibroblasts in the regulation of bone remodeling at tension side during OTM. More importantly, orthodontic force-treated primary fibroblasts from PDL showed the up-regulated expression of PDGFRβ and α-SMA in vitro than normal fibroblasts.

It is well known that PDGF-BB functions in directing mitogenesis, angiogenesis and osteogenic differentiation of osteoprogenitor cells or MSCs to enhance bone regeneration^14,15,32–34^. More importantly, PDGF-BB is FDA approved for use in the treatment of local periodontal defects and diabetic ulcers^35,36^. Bioinformatics analysis in this study identified that PDGF-BB/PDGFRβ signals were relevant to the activation of JAK/STAT3 signaling pathways, which was also consistent with our findings in OTM model. JAK2 and STAT3 displayed a slight increase and became significant at day 14 in periodontal ligament of tension side. STAT3, a latent transcription factor residing in the cytoplasm, is an important determinant of cell survival and proliferation^37^. Compared with other STATs, STAT3 has been demonstrated to participate in the maintenance of bone homeostasis in osteoblasts^38^. Previous research has reported that PDGF directly activates the JAK–STAT pathway and induces mitogens in fibroblasts^39^. Inactivation of STAT3 resulted in unresponsive osteoblasts to mechanical loading and limited bone formation in knock-out mice^40^. It was demonstrated that in vitro mechanical force upregulated the expression of Runx-2 gene in osteoblasts via potentiation of Polycystin-1 (PC1)-JAK2/STAT3 signaling axis, culminating to possibly control osteoblastic differentiation and ultimately bone formation^38^. It is well known that imatinib inhibit the expression and activation of PDGFRβ in vitro and in vivo by targeting the ATP-binding site of tyrosine kinases of PDGFRβ, thus preventing PDGFRβ autophosphorylation on PDGF-BB binding and inhibiting their biological effect^41,42^. AG490, also called tyrphostin, is a synthetic derivative of benzylidenemalononitrile. It is a specific and potent inhibitor of JAK2 signaling^43,44^. Therefore, we locally injected the inhibitors (imatinib and AG490) for PDGFRβ and JAK–STAT signals during OTM, and it was found that the treatment of imatinib and AG490 blocked the tooth movement compared with the Vehicle group by down-regulating the osteogenic differentiation and bone formation in tension side. Meanwhile, the expressions of PDGFRβ, JAK2 and STAT3 were decreased upon the administration of inhibitors compared the Vehicle group, which further demonstrated that PDGFRβ and JAK2/STAT3 signals was involved in bone formation in tension side during OTM.

Taken together, mechanistically, we suggested that PDL fibroblasts sensed and transduced the mechanical tensile force to the periodontal ligament and alveolar bone, and led to periodontal ligament remodeling and eventually alveolar bone formation through PDGF-BB/PDGFRβ/JAK2/STAT3 signaling pathway during OTM.

## Methods

### Orthodontic tooth movement model

80 6-week-old male Sprague–Dawley rats with a weight of 200 ± 20 g were used for in vivo experimental study, which were obtained from the Sino-British SIPPPR/BK Lab. Animal Ltd. All rats were housed under a 12/12 h day/night cycle with sterile food and water. They were adaptively fed for 7 days in the new environment before experiment. All animal experimental procedures were conducted in accordance with the regulations and guidelines of the Medical Ethics Committee of Nanjing Stomatological Hospital, Medical School of Nanjing University. The orthodontic tooth movement (OTM) model was performed as previously described^5,45^. In brief, the maxillary left first molar was moved mesially by ligation of a nickel-titanium closed-coil spring (3M Unitek, Monrovia, CA, USA) with 0.2 mm stainless steel ligatures between the upper incisors and the left first molar (Fig. 1A). Previous studies have demonstrated that a 40–60 g level of force stimulated substantial molar tooth mesial movement in rats^5,46,47^. A nickel-titanium spring was used to provide a relatively constant force of approximate 50 g during the course of the experiment. For the placement of the spring, grooves were drilled at the tooth cervix of the incisors and self-curing resin was boned on the incisors to prevent slipping. The right maxillary first molar without OTM served as the control.

Among them, 48 rats were subjected to volumetrically equivalent injections of 10 μL 1 mg/ml PDGFRβ inhibitor imatinib (Selleck, USA) or 5 mg/ml JAK/STAT inhibitor AG490 (MedChemExpress, USA), or phosphate-buffered saline (PBS) vehicle. All injections were administrated sub-periosteally into the distopalatal area of the left maxillary first molar using a 30-gauge needle (Hamilton Company, Reno, NV), every 2 days from the beginning of OTM^43^.

### Rat PDL fibroblasts isolation and culture

PDL fibroblasts were isolated from the extracted left maxillary first molar (Force group) and the right maxillary first molar (Control group) of SD rats at day 10 of OTM for in vitro study as described previously^8,30^. Briefly, gingivae of upper first molars were scraped off, and periodontal ligaments were digested with 3 mg/mL collagenase type I and 4 mg/mL dispase II for 1 h at 37 °C. Then the released single-cell suspensions were cultured in Dulbecco’s modified Eagle medium (DMEM, Gibco, USA) containing 10% fetal bovine serum (FBS, Gibco, USA). The medium was changed every 3 days until the cells reached 80% confluence. Cells (Control group and Force group) at passage 3–5 were used for further experiments. The experiment was performed in triplicate.

### Flow cytometry

Flow cytometry was used to characterize the orthodontic force-treated primary fibroblasts and normal fibroblasts from PDL. Briefly, PDL fibroblasts were digested and stained with antibodies against rat CD11b (FITC-conjugated; BioLegend, USA), rat CD45 (Alexa Fluor 647-conjugated; BioLegend, USA), rat CD29 (PE-conjugated; BioLegend, USA), rat CD90 (PE-conjugated; BioLegend, USA), rat PDGFRβ (PE-conjugated, BioLegend, USA) and the relative isotype control antibodies^48–50^. After incubation for 30 min at 37 °C in the dark, the samples were analyzed using the FACS Calibur flow cytometer (Becton Dickinson). The experiment was performed in triplicate.

### Sequential fluorochrome labeling

It is well known that tetracycline (TE) and Alizarin Red S (AL), as calcium chelating agents, can combine with calcium and deposit in the front of bone mineralization. The process of new bone formation and mineralization along the alveolar bone edge in the tension side of the maxillary first molar was assessed using a polychrome sequential fluorescent labeling method as we described previously^14,22,51^. Briefly, different fluorochrome were injected intraperitoneally at a sequence of 25 mg/kg Tetracycline Hydrochloride (Sigma, USA) and 30 mg/kg Alizarin Red S (Sigma, USA) in 16 rats at day 7 and day 14 of OTM, respectively.

### Histomorphometric observation

16 sequential fluorochrome labeled rats were sacrificed at 28 days of OTM, and then the maxillary jaws were fixed in 10% buffered formalin, as previously described^15,19^. The specimens were dehydrated in ascending concentrations of alcohol from 75 to 100% and embedded in polymethyl-methacrylate. After sclerosis, the specimens were cut along the transverse plane (n = 8/group) or sagittal plane (n = 8/group) into 150-μm-thick sections using a microtome (Leica, Germany). After sectioning, they were grounded and polished to final 40-μm-thick sections for fluorescence labeling observation under confocal laser scanning microscope (OLYMPUS, Japan) or staining with van Gieson's picro fuchsin for histological analysis, as previously described^15,52^. Excitation/emission wavelengths of chelating fluorochromes were used 405/580 nm and 543/617 nm for Tetracycline Hydrochloride (green) and Alizarin Red S (red), respectively^51^.

### Micro-CT analysis of alveolar bone

The rats were sacrificed with an intraperitoneal overdose injection of chloral hydrate at day 7 and day 14 (n = 8, respectively). The amount of tooth movement was defined as the distance from the most mesial point of the maxillary second molar to the most distal point of the first molar measured by an electronic caliper, which was accurate to two decimal places. Each distance was measured three times by the same observer, and the mean was used. The maxillae were then obtained and fixed in a 4% phosphate-formalin solution for 24 h. In order to study the changes of alveolar bone microstructure during OTM, the maxillae were subjected to Micro-computed tomography (Micro-CT) system (Bruker microCT, Kontich, Billerica, MA, USA), as previously reported^53^. Computed tomography was conducted using a slice thickness of 18-μm, voltage of 50 kV, and electrical current of 455 μA. After scanning, three-dimensional images were reconstructed by the Bruker micro-CT version 1.1. The region of interest (ROI) was defined as the zone of alveolar bone at the distal coronal one-third area of the distal root (the tension area, T) and the mesial coronal one-third area (the pressure area, P). To obtain image gray values as a measure of bone mineral density (BMD), two standard samples with known BMD values (0.25 and 0.75 g/cm^3^) were employed for calibration against their attenuation coefficients. For quantitative analysis, all values were assessed within ROI to calculate the following parameters: bone volume/tissue volume (BV/TV, %), trabecular number (Tb.N, 1/mm), trabecular thickness (Tb.Th, mm), and trabecular separation (Tb.Sp, mm).

### Histology and immunohistochemistry analysis

After micro-CT scanning, samples were decalcified with 10% EDTA for 8 weeks, and then embedded in paraffin. Consecutive 4-μm paraffin sections were obtained from the sagittal direction. All histological sections were scanned by an automatic digital slide scanner (3DHISTECH, Hungary) and then observed with Case Viewer 2.3 (3DHISTECH, Hungary). After dewaxing and rehydration, some sections were stained with haematoxyl and eosin. And some sections were subjected to immunohistochemical analysis incubated with anti α-SMA, anti-Runx-2, anti-OCN (Proteintech, China) and anti-PDGF-BB, anti-PDGFRβ, anti-JAK-2, anti-STAT3 antibodies (Abcam, USA) overnight at 4 °C. Then, sections were incubated with secondary antibody (DAKO, Glostrup, Denmark) for 1 h at room temperature. Finally, the samples were stained with Diaminobenzidine (DAB) substrate (DAKO, USA) and lightly counterstained with hematoxylin. For the semi-quantitative evaluation, the scoring method was applied, as described previously^54^. Two experienced “blinded” readers scored the tension area in the slides according to the percentage of positive cells: 0 points implied less than 10% positive cells, 1 point implied 11–25% positive cells, 2 points implied 26–50% positive cells, 3 points implied 51–75% positive cells, and 4 points implied greater than 75% positive cells. Staining intensity was defined as 0, negative staining; 1, weak staining; 2, moderate staining and 3, strong staining. Final immunohistochemical scores were calculated by multiplying the percentage score and the intensity score^55^.

### TRAP staining

For tartrate-resistant acid phosphatase (TRAP) staining, paraffin sections were soaked in TRAP dye (Wuhan Goodbio Technology Co., Ltd., China). Osteoclasts were detected as wine-red multinuclear cells after (TRAP) staining. TRAP-positive multinucleated osteoclasts were counted in the pressure area^51^.

### Immunofluorescence assay

Immunofluorescence assay was performed as previously described^19,56^. Briefly, PDL fibroblasts were fixed with 4% formaldehyde for 30 min at 4 °C. Then fibroblasts were permeabilized with 100% methanol at − 20 °C and blocked with 3% bovine serum albumin and was incubated with primary antibodies against α-SMA (Abcam, USA) and PDGFRβ (Abcam, USA) for fibroblasts overnight at 4 °C. The samples were then incubated with red and green fluorescent labeled secondary antibodies in the dark at 37 °C. Next, the nuclei were stained with DAPI (Bioword, China). Finally, the specimens were observed using the confocal laser-scanning microscope (OLYMPUS, Japan).

### Bioinformatics analysis

We adopted bioinformatics analysis to explore the biologic characteristics of PDGFRβ shared by (Search Tool for the Retrieval of Interacting Genes database) STRING (version 11.0; https://string-db.org/). STRING is intended to assess the functional protein association networks^57,58^. Biological interpretations of genes have been typically evaluated by the Gene Ontology (GO) and Kyoto Encyclopedia of Genes and Genomes (KEGG) databases^59^. The KEGG PATHWAY database is a compilation of manually verified maps of biological interactions represented by the complete set of pathways related to signal transduction and other cellular processes. The biological process (GO) focused on “response to stress”, and the KEGG pathway predicted the potential PDGFRβ related signaling pathway.

### Statistical analysis

Statistical comparisons were carried out via one-way analysis of variance with Tukey’s post hoc test^60^. All the data were presented as mean ± standard deviation. *P* < 0.05 among the various groups was considered statistical significance. All statistical analysis was carried out by the SPSS 18.0 statistical software package.

### Ethics statement

All-animal experimental procedures were approved by the Medical Ethics Committee of Nanjing Stomatological Hospital, Medical School of Nanjing University, and the ethics approval number was 2019NL-009(KS). All experiments involving rats were performed in accordance with relevant guidelines and regulations.

## Data availability

The datasets generated during and/or analyzed during the current study are available from the corresponding author on reasonable request.
